# Is Oxidative Stress an Emerging Player in the Thrombosis of Patients with Anti-Phosphatidylethanolamine Autoantibodies?

**DOI:** 10.3390/jcm11051297

**Published:** 2022-02-27

**Authors:** Xavier Heim, Daniel Bertin, Noémie Resseguier, Abdelouahab Beziane, Audrey Metral, Alexandre Brodovitch, Régis Guieu, Jean-Guillaume Steinberg, Marcel Blot-Chabaud, Pierre-Emmanuel Morange, Jean-Louis Mege, Nathalie Bardin

**Affiliations:** 1 Service d’Immunologie, Pôle de Biologie, Hôpital de la Conception, Assistance Publique-Hôpitaux de Marseille (AP-HM), 13005 Marseille, France; daniel.bertin@ap-hm.fr (D.B.); abdelouahab.beziane@ap-hm.fr (A.B.); audrey.metral@free.fr (A.M.); alexandre.brodovitch@ap-hm.fr (A.B.); jean-louis.mege@univ-amu.fr (J.-L.M.); nathalie.bardin@univ-amu.fr (N.B.); 2 Center for CardioVascular and Nutrition Research C2VN, INSERM, INRAE Faculty of Pharmacy, Timone Campus, Aix-Marseille University, 13005 Marseille, France; regis.guieu@univ-amu.fr (R.G.); jean.steinberg@anciens.univ-amu.fr (J.-G.S.); marcel.blot-chabaud@laposte.net (M.B.-C.); pierre-emmanuel.morange@univ-amu.fr (P.-E.M.); 3 Service de Santé Publique, Unité de Recherche EA 3279, Aix-Marseille Université, 13005 Marseille, France; noemie.resseguier@univ-amu.fr; 4 Service de Biochimie, Pôle de Biologie, Hôpital de la Timone, Assistance Publique-Hôpitaux de Marseille (AP-HM), 13005 Marseille, France; 5 Service d’Hématologie, Pôle de Biologie, Hôpital de la Timone, Assistance Publique-Hôpitaux de Marseille (AP-HM), 13005 Marseille, France; 6 Institut Hospitalo-Universitaire (IHU) Méditerranée Infection, Aix-Marseille Université, 13005 Marseille, France

**Keywords:** reactive oxygen species (ROS), thrombosis, anti-phosphatidylethanolamine autoantibodies, ELISA, antiphospholipid syndrome, thiobarbituric acid-reactive substances (TBARs)

## Abstract

The detection of anti-phosphatidylethanolamine autoantibodies (aPEs) has been proposed to improve the diagnosis and management of patients presenting clinical manifestations of antiphospholipid syndrome (APS), such as thrombosis, and who are persistently negative for conventional markers. After selecting the most specific ELISA for their detection, we evidenced the interest of aPEs in the exploration of thrombosis when APS conventional markers were negative through a 1-year retrospective study including 1131 consecutive patients routinely tested for aPEs. To validate this result, we assessed aPEs in a newly selected population of 77 patients with unexplained deep vein thrombosis (DVT). With a total prevalence of 19.5%, we confirmed the interest of aPE detection in patients with unexplained DVT who were devoid of other aPLs markers. Since endosomal compartment, a source of ROS production, has been recently identified as the cellular target of aPEs in vitro, we then investigated an association between aPE positivity and reactive oxygen species (ROS) production by measuring the production of thiobarbituric acid-reactive substances. We showed, for the first time, a significant association between aPE positivity and systemic ROS production in patients which led us to hypothesize a new mechanism of action of aPEs in thrombosis through a signaling related to oxidative stress.

## 1. Introduction

The detection of autoantibodies directed against phosphatidylethanolamine (aPE) has been proposed to improve the diagnosis and management of patients presenting clinical manifestations of antiphospholipid syndrome (APS), such as thrombosis and/or obstetric diseases, and who are persistently negative for conventional markers including lupus coagulant (LA), IgG and/or IgM anticardiolipin autoantibodies (aCL) and IgG and/or IgM anti β2 glycoprotein I autoantibodies (aβ2GPI). Although numerous antiphospholipid autoantibodies (aPL) target anionic phospholipid, aPEs are directed against a zwitterionic neutral phospholipid made up of a glycerol molecule esterified by two fatty acids and a phosphoethanolamine residue. PE is a dominant aminophospholipid of most living organisms, asymmetrically distributed in the biological membrane and mainly located in its inner leaflet [1,2,3,4]. PE is involved in cellular processes such as cell fusion, cell cycle, and autophagy [5,6,7,8,9]. In fact, aPEs have been proposed to be associated with a procoagulant state which support their interest as markers in the thrombotic context [10]. In a multicentric study, the prevalence of aPEs in patients with unexplained thrombosis was 15%, but only 3% in controls (*p* < 0.0001) [11]. Several studies highlighted the positivity of aPEs in thrombotic events, even in the absence of conventional antiphospholipid autoantibodies (aPL), reinforcing their potential interest as diagnostic markers. Although there is a growing interest in aPEs, only a few clinical laboratories routinely test their presence. One reason for that is the lack of specificity of the commercially available ELISA kits to aPEs, as they also detect the human co-factor B2-glycoprotein I (B2GPI). Concerning the mechanism of action of these autoantibodies, it has recently been shown that early endosomes constitute their cellular target [12]. Since endosomes are of key importance for protein intracellular trafficking, it is suggested that aPEs could have a broad impact on different types of cells. These autoantibodies could especially contribute to the dysregulation of membrane redox enzymes and stimulate reactive oxygen species (ROS)-mediated signaling pathways. Oxidative stress is recognized as an important mediator of thrombotic events [13,14]. Therefore, it is relevant to assess ROS production in patients positive for aPEs. However, owing to its short half-life, the direct quantification of ROS is considered challenging. In contrast, ROS byproducts such as thiobarbituric acid-reactive substances (TBARs) which are formed by the peroxidation of lipids, are more stable and can be detected [15]. TBARs measure the global effects of ROS production, and more importantly the effects of tissue damage, and its level is influenced by the ratio of oxidizing/pro-oxidizing agents [16].

The aim of the present study was thus to investigate the clinical interest of aPE detection using a specific in-house-developed ELISA, and to analyze the potential association between aPE positivity and ROS production in patients with unexplained deep vein thrombosis. 

## 2. Materials and Methods

### 2.1. Patients

To compare the efficiency between the in-house-developed and commercially available ELISAs for specifically detecting aPEs, we used sera from 67 patients whose samples were sent to our department at the Immunology Laboratory of Conception Hospital (AP-HM, Marseille, France) for the routine detection of IgG-aPE and IgM-aPE autoantibodies in addition to other conventional aPL. The sera were stratified as follows: 23 IgG-aPE positive (18 IgG/IgM-aB2GPI negative and 5 IgG-aB2GPI positive), 26 IgG/IgM-aPE negative (13 IgG-aB2GPI positive and 13 IgM-aB2GPI positive) and 18 IgM-aPE positive (all IgG/IgM-aB2GPI negative). The average age of patients was 50.1 ± 18 years, of which 18 were male and 49 were female.

A retrospective study was also performed on 2255 patients who had their aPE antibodies quantified at the Immunology Laboratory of Conception Hospital (AP-HM, Marseille, France) over a period of 1 year. Among these, 1124 patients were excluded from the study because of the lack of their clinical and biological data.

Therefore, the size of the population enrolled in the study reached 1131 patients. The collected clinical and biological data were the following: age, sex, medical departments, dosage of IgG- and IgM-anti-B2GPI, IgG- and IgM-aCL, platelet count, number of thromboses and their characteristics (recurrence, type of thrombosis), and obstetric complications (fetal loss, postpartum hemorrhage, pre-eclampsia, and infertility).

Arterial thromboses included: myocardial infarction, stroke, or any other acute thrombotic event involving the arterial vascular system.

Venous thromboses included: deep vein thrombosis, pulmonary embolism, or any other acute thrombotic event involving the venous vascular system.

An event was considered as recurrent when the number of events was greater than or equal to 2. 

To confirm the interest of quantifying IgG- and IgM-aPE antibodies in patients with clinical symptoms of APS in the absence of conventional autoantibodies, a cohort of 77 patients with deep venous thrombosis (DVT) who were negative for conventional antiphospholipid autoantibodies were selected after careful clinical examination and normal biological screening including prothrombin time (PT), activated partial thromboplastin time (aPTT), D-dimer, fibrinogen, factor VIII level, lupus anticoagulant (LA), anti-cardiolipin, and anti-β2 glycoprotein I antibodies. LA was assessed using two clotting times: partial thromboplastin time–lupus anticoagulant (PTT-LA) and diluted Russel viper venom time (dRVVT). Inherited thrombophilia etiologies were also ruled out by researching antithrombin (AT), protein C (PC) and protein S (PS) deficiencies, factor V Leiden (FVL), and the G20210A prothrombin mutation (PTM).

All samples were taken from a declared Biobank (DC 2012-1704) in compliance with ethical directives. This study was approved and registered by the Assistance Publique Hôpitaux de Marseille and fulfilled local requirements in terms of data collection, patients’ consent, and protection of data (RGPD/APHM 2019-108). This study was conducted according to the Declaration of Helsinki.

### 2.2. aPE Detection

#### 2.2.1. In-House ELISA with PE-Only Coating

Irradiated polystyrene microtiter plates (Maxisorp^®^, Nunc^®^, Rockilde, Denmark) were coated (30 µL/well) either with a solution containing 50 µg/mL PE from egg yolk (Sigma-Aldrich, Saint-Quentin-Fallavier, France) or with the solvent absolute ethanol (>99.7%, VWR Chemicals Prolabo), used to determine the non-specific binding of each sample. The plates were dried by evaporation at 18 °C and blocked by incubating with 10% fetal calf serum (FCS, GIBCO^®^, Invitrogen, Cergy-Pontoise, France) in PBS for 1h at room temperature (RT). Finally, plates were dried using nitrogen gas.

Sera were diluted 1:100 in 10% FCS-PBS and added in duplicate to coated and non-coated wells (100 µL/well) and incubated for 1h at RT. After the washing step (three times with PBS), 100 µL of alkaline-phosphatase-conjugated goat anti-human immunoglobulin (Jackson ImmunoResearch Laboratories, West Grove, PA, USA) IgG (diluted 1:5000 in PBS-FCS) or IgM (diluted 1:2400 in PBS-FCS) were added in each well. Following 1h incubation at RT and the washing step, a phosphatase substrate para-nitrophenylphosphate, pNPP (Sigma-Aldrich, Saint-Quentin-Fallavier, France) (1 mg/mL in 1M diethanolamine buffer, 0.5 mM MgCl_2_, pH = 9.8) was added (100 µL/well) and the plate was incubated at 37 °C for 15 min (IgG-aPE) or 20 min (IgM-aPE). Then, optical density (OD) was measured at 405 nm. OD for each sample was obtained by subtracting the OD of the non-coated well from that with antigen. The cut-off levels for IgG-aPE and IgM-aPE were determined with 100 sera from age- and sex-matched healthy blood donors, corresponding to the 99th percentile of control group values. These cut-off values were 0.47 and 0.68 OD, corresponding to the following arbitrary units: 18 and 59 U/mL for IgG-aPE and IgM-aPE, respectively.

#### 2.2.2. Commercial aPE ELISA with PE and B2GPI Coating

We used the commercial kit provided by Theradiag (Marne-la-Vallée, France) and manufactured by Aesku (Wendelsheim, Germany). As mentioned by the manufacturer and highlighted in the datasheet, AESKULISA Ethanolamine-GM is a solid-phase enzyme immunoassay coated with purified phosphatidylethanolamine and native human B2GPI proteins.

### 2.3. Adsorption Experiments on B2GPI Coated Plate

The adsorption experiments were carried out as follows. Briefly, sera were selected for IgG-aB2GPI positivity as confirmed by the IgG-aB2GPI ELISA kit from Orgentec. Two hundred microliters of diluted patient’s sera (1:100) were added to 1µg B2GPI-coated plate (human β_2_-glycoprotein, the binding site) and incubated for 1h at RT. After the first adsorption, a second adsorption was carried out by the same procedure and this adsorption procedure was repeated identically up to four times. The efficiency of the adsorption procedure was verified by the IgG-aB2GPI assay on the sera processed before and after the adsorption experiments.

### 2.4. TBARs Dosage

Serum TBARs concentration was assessed according to Uchiyama and Mihara method [15] modified by Jammes [17]. After the centrifugation of blood samples (1500× *g* at 4 °C for 10 min), 300 µL of serum was deproteinized in 10% trichloroacetic acid (TCA, v/v) containing 2.90 mmol of ethanolicbutylatedhydroxytoluene (Sigma-Aldrich, Saint-Quentin Fallavier, France) to avoid further peroxidation. After vortexing and centrifugation (2500× *g* at 4 °C for 15 min), the supernatants were stored at −80 °C until use. Samples were then aliquoted (200 µL) and 200 µL of 8.1% sodium dodecylsulfate, 1.5 mL of 20% acetate buffer (pH = 3.5), 1.5 mL of freshly prepared 0.8% thiobarbituric acid (Sigma-Aldrich Co.) and 600 µL of water were then successively added. The test tubes containing glass beads were heated at 100 °C for 60 min and cooled with tap water. Then, 4 mL of n-butanol and 1 mL of water were added into each tube. After vortexing for 5 min, the mixture was centrifugated (2000× *g* for 3 min) to obtain a rapid separation between organic and aqueous phases. The upper organic phase was pipetted off and the pink pigment was measured using a spectrofluorimeter at an excitation wavelength of 515 nm and an emission wavelength of 553 nm (Shimadzu model RF5000; Shimadzu, Kyoto, Japan). A standard curve of TBARs was obtained after overnight hydrolysis in a solution containing 1 mmol of tetraethoxypropane (Sigma-Aldrich Co.) in 0.1 N HCl at RT.

### 2.5. Statistical Analysis

Statistical analysis comparing the positivity or negativity of IgG and IgM aPE with the presence or absence of the other studied parameters was performed using the Chi-2 method. The concordance between the in-house-developed and commercial aPE ELISA was evaluated using Cohen’s Kappa coefficient, which takes on the value: zero if there is no more agreement between two tests than expected by chance; 1 if there was a perfect agreement. Kappa values below 0.4 were considered as poor agreement. Paired sample *t*-test was used to compare aPE ELISA optical densities before and after serum adsorption on B2GPI. Student’s *t*-test was used to compare the mean values of TBARs levels. Dot plots, box plots and statistical calculations were performed with GraphPad Prism version 5.03 (GraphPad Software, La Jolla, CA, USA). The threshold for statistical significance was set at *p* = 0.05. Effect size (Cohen’s d) and power value were calculated using pwr library of R software version 3.6.0 (R Foundation for Statistical Computing).

## 3. Results

### 3.1. Specific Detection of aPE

**Figure 1:**
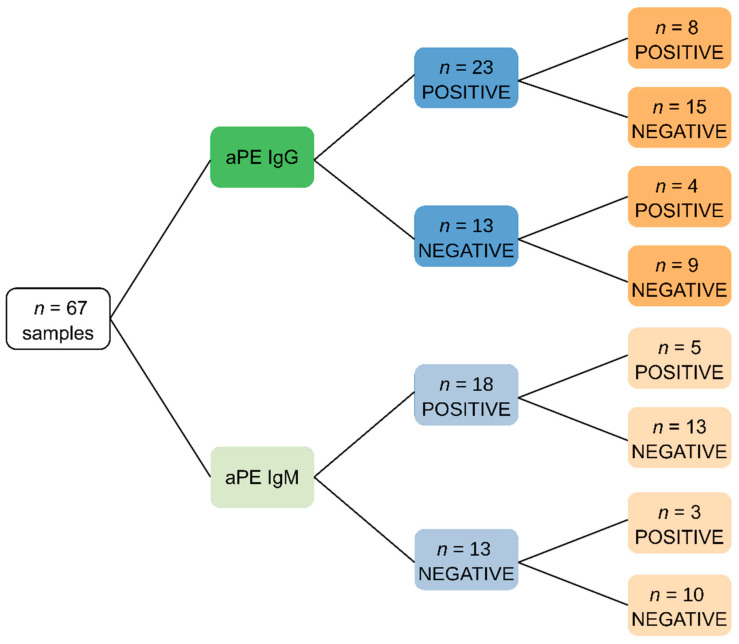
Tree representation of results obtained using different ELISA assays. Tree diagram showing serological status of patients towards phosphatidylethanolamine according to in-house or commercial aPE ELISA. aPEs: anti-phosphatidylethanolamine autoantibodies.

**Figure 2:**
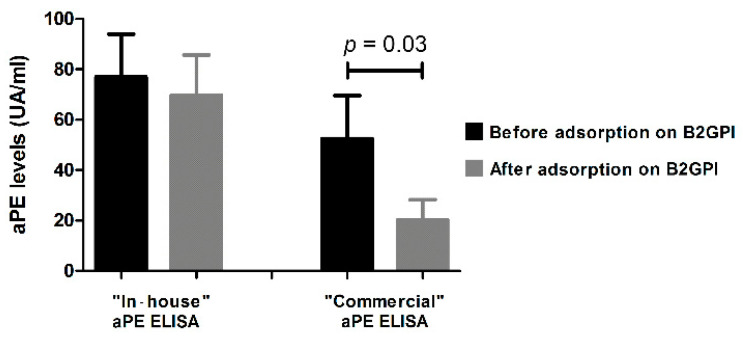
Comparison of measured optical densities (OD) between in-house and commercial aPE ELISA. Comparison of measured optical densities (OD) between in-house and commercial IgG aPE ELISA. Measurements were performed before and after adsorption of sera on immobilized beta-2-glycoprotein I (B2GPI). Adsorption did not significantly decrease the measured OD with the in-house ELISA, whereas OD was significantly decreased with the commercial aPE ELISA (*p* = 0.03, paired samples *t*-test). Bars represents means and whiskers represents SEM. aPE: anti-phosphatidylethanolamine autoantibodies.

To check ELISA specificity for aPE detection, we first compared our in-house ELISA to the commercial one. To this end, sera from 67 patients explored in the routine practice were analyzed. From the 23 patients who tested positive for IgG-aPE in the in-house ELISA, only 8 sera were positive when tested using the commercial kit. Besides, among the 18 IgM-aPE positive sera, only 5 were found to be positive using the commercial kit (Figure 1). Accordingly, the kappa coefficient was 0.042, showing poor concordance between the two methods. Since human B2GPI was coated with PE in the commercial ELISA, adsorption experiments were conducted on immobilized B2GPI to further explore discrepancies between the two ELISA systems (Figure 2). Five positive sera with the in-house ELISA (three positive and two negative with the commercial ELISA) were tested with aPE ELISA before and after the adsorption test. All of them were aB2GPI positive. After the adsorption procedure, their reactivity towards PE significantly decreased in the commercial ELISA but not with the in-house aPE ELISA. This shows that the reactivity obtained with commercial kit could be partly related to a reactivity against the B2GPI, excluding its use for specific aPE detection.

### 3.2. Retrospective Clinical Study 

Sera from a total of 1131 patients including 848 women (75%) and 283 men (25%), with a mean age of 46 ± 16 years were used to quantify aPE antibodies using the in-house-developed ELISA. Patients’ serum samples were addressed to our laboratory by departments of neurology (35.2%), internal medicine (32.6%), thrombosis exploration (15.1%), dermatology (8.8%), hematology (2.7), infectious diseases (2.4%), gynecology (1.2%), nephrology (1.1%), post-emergency medicine (1.1%) and others (intensive care, gastro-enterology, cardiology, rheumatology, surgery, otorhinolaryngology, addictology, odontology, dermatology, psychiatry, oncology). Geologically, all patients were from southeast France.

Clinical and biological data collected from all patients (Table 1) revealed that 25.7% of patients had at least one thrombosis, 21.0% of women had at least two fetal losses, 2.9% infertility, and 3.5% had other obstetrical complications. All conventional aPL markers were assessed and were of low prevalence. In contrast, the prevalence in patients with aPE, (IgG and/or IgM) was 20.7% (234/1131), of which 89% had IgG-aPE (209/234) and 16% had IgM-aPE (38/234). Among them, 99 patients positive for aPEs had an additional dosage at least 12 weeks after they were observed. This allowed us to measure antibody persistence rate and we show it to be 67% (33/99).

As presented in Table 2, an association between aPEs and thrombosis was near statistical significance (*p* = 0.06), but no clear association with fetal loss (*p* = 0.83), or other obstetrical complications (*p* = 0.12) were obtained. We also noted a trend towards statistical significance between the association of aPEs and infertility (*p* = 0.06). Clinical association was further analyzed according to the aPE isotype. No significant association was found between IgM-aPE and all the clinical contexts described in Table 2. In contrast, we found a significant association between IgG-aPE prevalence and thrombosis (*p* = 0.04) but no association with obstetrical manifestations (Table 2). No preferential association between IgG aPE and type of thrombosis (arterial or venous, cerebral stroke, pulmonary embolism, myocardial infarction) was found. 

Among these IgG aPE-positive patients with a history of thrombosis, 34% (22/65) were positive for conventional aPL, and the majority of them (66%, 43/65) had unexplained thrombosis without conventional aPL markers. 

In this subgroup of patients without conventional aPL, we found 24 patients with deep vein thrombosis, 12 strokes, and 9 with pulmonary embolism. A total of 26 patients had isolated thrombosis and 17 patients had recurrent thrombosis.

**Figure 3:**
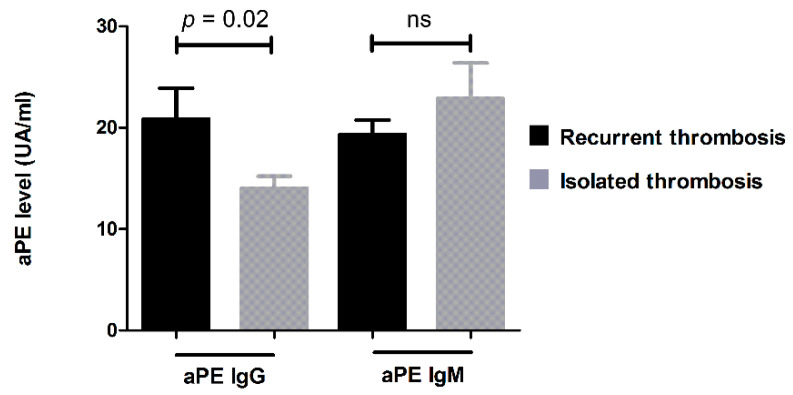
Quantitative study of aPEs in thrombotic patients. Comparison of aPE levels for each isotype in patients suffering from isolated or recurrent thrombosis. aPEs: anti-phosphatidylethanolamine autoantibodies; ns: not significant. Bars represents means and whiskers represents SEM.

Interestingly, we found a significant correlation between the level of IgG-aPE and the presence of thrombosis (*p* = 0.03). The higher the level of IgG-aPE, the greater the risk of developing thrombosis. Moreover, the level of IgG-aPE was significantly associated with the recurrence of thrombotic events (*p* = 0.02). (Figure 3).

### 3.3. Association between aPEs and Oxidative Stress in Patients with Unexplained DVT

**Figure 4:**
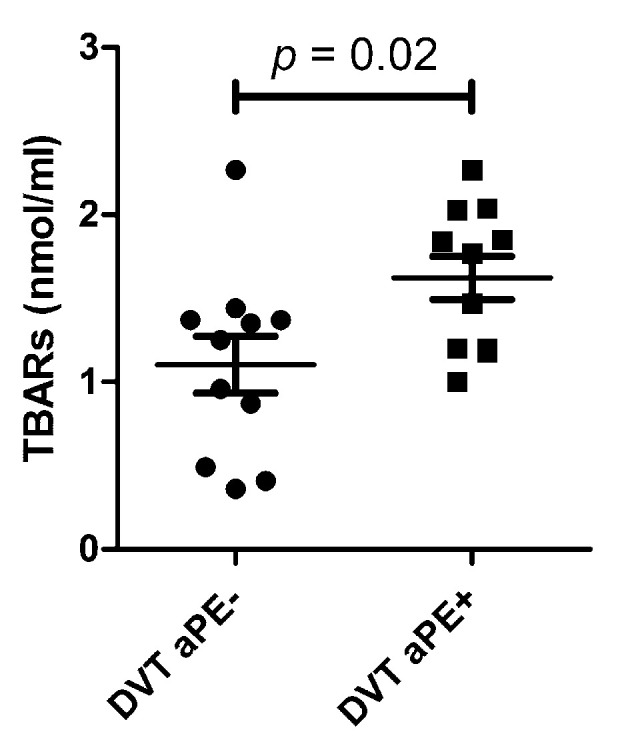
Comparison of TBARs levels in sera of patients with deep vein thrombosis. Comparison of TBARs levels in sera of patients with deep vein thrombosis (DVT) according to their aPE serological status. The largest horizontal line shows the mean value, and whiskers represent SEM. Serum levels of TBARs were significantly higher in aPE+ DVT patients than aPE− DVT patients (*p* = 0.02, unpaired *t*-test). TBARs: thiobarbituric acid-reactive substances; aPEs: anti-phosphatidylethanolamine autoantibodies; DVT: deep vein thrombosis.

As it has been reported that aPEs could stimulate the ROS pathway, we investigated whether the production of ROS was associated with aPE positivity. As unexplained thrombosis is one of the most prevalent clinical contexts associated with aPE positivity, we explored 77 patients with unexplained DVT, all negative for conventional aPL. We found a total prevalence of 19.5% for aPEs (14.3% IgG-aPE and 5.2% IgM-aPE) which confirms the results obtained from the retrospective study. We then measured the concentration of TBARs in the serum of patients with DVT. According to the availability of the samples, a total of 22 samples were tested. In these experiments, the concentration of TBARs informed the systemic level of lipoperoxydation resulting from ROS production. As shown in Figure 4, the concentration of TBARs was significantly higher in thrombotic aPE-positive patients than in those negative for aPEs (effect size and power value were 1.04 and 0.637, respectively).

## 4. Discussion

The interest of using aPEs as biomarkers has been poorly studied in the literature and actions for their detection are not systematically performed in routine clinical practice. In this study, we evidenced the interest of aPEs in the exploration of APS, especially when conventional markers were negative, using the results obtained from 1131 unselected patients and 77 selected patients with unexplained DVT. We showed, for the first time in patients with unexplained thrombosis, that oxidative stress was higher in aPE-positive than in aPE-negative patients, raising an interesting hypothesis on the mechanism of action of aPE in thrombosis. 

Other studies have highlighted the importance of testing aPEs in cases of thrombosis [10,11]. In particular, results concerning prevalence and isolated positivity of aPEs (without conventional aPL) are in accordance with our findings. For instance, Sanmarco et al. [10] found a significantly higher prevalence of aPE (18%) in patients with unexplained thrombosis. In the same way, Sanmarco et al. showed that aPEs were the aPL with the highest odd ratio for thrombosis ([OR]: 4.2; *p* = 0.001) [11]. In our study, we additionally found a significant association between the level of IgG aPE and the recurrence of the thrombotic event. Although studies had previously shown an association between aPEs and obstetric complications [18], such an association was not observed in our study performed on a routine population of unselected patients. In addition, we found an association with a trend towards statistical significance between aPEs and infertility. This result is in agreement with a prospective study that studied the prevalence of aPL in 101 women who underwent in vitro fertilization, and showed that aPEs were the most prevalent aPL [19].

Until now, PE has appeared as a major anticoagulant factor involved in the anticoagulant C protein pathway. In fact, it has been suggested that the inhibitory effect of aPEs on this anticoagulant pathway is one of the mechanisms leading to thrombosis [20,21,22]. However, the patients enrolled in our study had normal protein C activity. This apparent discrepancy may be explained by the fact that PC activity is an in vitro chronometric assay performed in the presence of an excess of phospholipids, which prevents aPEs from interfering with PC activity measurement. 

Here, we showed for the first time that aPE-mediated thrombotic effects are regulated through ROS signaling. Indeed, we found that TBARs production was significantly higher in aPE-positive thrombotic patients than in negative ones. Intracellular ROS such as superoxide anion and hydrogen peroxide are directly involved in platelet activation and thrombus formation [13,14]. Thus, the overproduction of ROS may be associated with diseases such as hypercholesterolemia, diabetes, hypertension and metabolic syndrome, all risk factors for thrombosis. Hou et al. demonstrated that PE antigen may be accessible in early endosomes, by using different PE-binding agents including plasma from an aPE- positive patient and purified aPEs [12]. The authors suggested that these autoantibodies could act through ROS generation. This agrees with our results obtained from thrombotic patients, showing that aPEs are involved in ROS production, which themselves contribute to thromboses. In this study, we also emphasized the relevance of using highly specific aPE-detection ELISA kits. Indeed, our experiments show that with the commercially available ELISA assays, a great number of positive patients can be attributed to the presence of confounding antibodies directed against B2GPI. The development of commercial ELISA kits specific for PE is thus required. 

In conclusion, we demonstrated that aPEs should be assessed in thrombotic patients, especially when other aPLs markers are absent. The detection must be performed by using an ELISA with PE as the sole coating antigen. Finally, we show for the first time an association between aPE positivity and the systemic effect of ROS generation, suggesting a new mechanism of action of aPEs through ROS signaling in the thrombotic process. Studies are now necessary to confirm this concept in a larger cohort of patients and pave the way for investigating novel therapeutic strategies. 

## Figures and Tables

**Table 1:** Clinical and biological data of the population tested for aPEs.

Clinical or Biological Parameters	Number of Positive Patient for Each Parameter	Size of Population (Nb of Patients)	Prevalence
**Clinical events**			
-Thrombosis:	291	1131	0.257
-Arterial thrombosis	143	291	0.491
-Venous thrombosis	181	291	0.622
-Cerebral stroke	92	291	0.316
-Pulmonary embolism	76	291	0.261
-Myocardial infarction	12	291	0.041
-Recurrent fetal losses	178	848	0.21
-Obstetrical complications	30	848	0.035
-Infertility	25	848	0.029
**Biological data**			
aPE IgG and/or IgM	234	1131	0.207
aPE IgG	209	1131	0.185
aPE IgM	38	1131	0.034
aB2GPI IgG	44	1131	0.039
aB2GPI IgM	41	1131	0.036
aCL IgG	60	1131	0.053
aCL IgM	84	1131	0.074
LA	66	968	0.068

**Table 2:** Clinical events according to aPE serological profile.

	aPE IgG and/or IgM			aPE IgG			aPE IgM		
	− Nb (%)	+ Nb (%)	*p* value	− Nb (%)	+ Nb (%)	*p* value	− Nb (%)	+ Nb (%)	*p* value
Thrombosis	220 (24.5%)	71 (30.3%)	0.06	226 (24.5%)	65 (31.1%)	0.04	281 (25.7%)	10 (26.3%)	0.93
Recurrent pregnancy loss	139 (20.8%)	39 (21.6%)	0.83	146 (21.2%)	32 (20.1%)	0.76	169 (20.7%)	9 (28.1%)	0.31
Obstetrical complications	27 (4.1%)	3 (1.7%)	0.12	27 (3.9%)	3 (1.9%)	0.21	30 (3.7%)	0 (0%)	0.62
Infertility	16 (2.4%)	9 (5.0%)	0.06	17 (2.5%)	8 (5.0%)	0.11	23 (2.8%)	2 (6.3%)	0.24

aPEs: anti-phosphatidylethanolamine autoantibodies, Nb: number of patients.

## Data Availability

The data used to support the findings of this study are available from the corresponding author upon request.

## References

[1] Langley K.E., Kennedy E.P. (1979). Energetics of rapid transmembrane movement and of compositional asymmetry of phosphatidylethanolamine in membranes of Bacillus megaterium. Proc. Natl. Acad. Sci. USA.

[2] Li Z., Wells C.W., North P.E., Kumar S., Duris C.B., McIntyre J.A., Zhao M. (2011). Phosphatidylethanolamine at the Luminal Endothelial Surface—Implications for Hemostasis and Thrombotic Autoimmunity. Clin. Appl. Thromb. Hemost.

[3] Zachowski A. (1993). Phospholipids in animal eukaryotic membranes: Transverse asymmetry and movement. Biochem. J..

[4] Zwaal R.F., Roelofsen B., Comfurius P., van Deenen L. (1975). Organization of phospholipids in human red cell membranes as detected by the action of various purified phospholipases. Biochim. Biophys. Acta.

[5] Deeba F., Tahseen H.N., Sharad K.S., Ahmad N., Akhtar S., Saleemuddin M., Mohammad O. (2005). Phospholipid diversity: Correlation with membrane–membrane fusion events. Biochim. Biophys. Acta.

[6] Verkleij A.J., Leunissen-Bijvelt J., de Kruijff B., Hope M., Cullis P.R. (1984). Non-Bilayer Structures in Membrane Fusion. Ciba Found. Symp..

[7] Emoto K., Kobayashi T., Yamaji A., Aizawa H., Yahara I., Inoue K., Umeda M. (1996). Redistribution of phosphatidylethanolamine at the cleavage furrow of dividing cells during cytokinesis. Proc. Natl. Acad. Sci. USA.

[8] Hailey D.W., Rambold A.S., Satpute-Krishnan P., Mitra K., Sougrat R., Kim P.K., Lippincott-Schwartz J. (2010). Mitochondria Supply Membranes for Autophagosome Biogenesis during Starvation. Cell.

[9] Nebauer R., Rosenberger S., Daum G. (2007). Phosphatidylethanolamine, a Limiting Factor of Autophagy in Yeast Strains Bearing a Defect in the Carboxypeptidase Y Pathway of Vacuolar Targeting. J. Biol. Chem..

[10] Sanmarco M., Alessi M.-C., Harle J.R., Sapin C., Aillaud M.-F., Gentile S., Juhan-Vague I., Weiller P.-J. (2001). Antibodies to phosphatidylethanolamine as the only antiphospholipid antibodies found in patients with unexplained thromboses. Thromb. Haemost..

[11] Sanmarco M., Gayet S., Alessi M.-C., Audrain M., de Maistre E., Gris J.-C., de Groot P.G., Hachulla E., Harlé J.-R., Sié P. (2007). Antiphosphatidylethanolamine antibodies are associated with an increased odds ratio for thrombosis. A Multicenter Study with the Participation of the European Forum on Antiphospholipid Antibodies. Thromb. Haemost..

[12] Hou S., Fölsch H., Ke K., Mills J.C., Ramsey-Goldman R., Zhao M. (2017). Early endosome as a pathogenic target for antiphosphatidylethanolamine antibodies. Proc. Natl. Acad. Sci. USA.

[13] Qiao J., Arthur J.F., Gardiner E.E., Andrews R.K., Zeng L., Xu K. (2018). Regulation of platelet activation and thrombus formation by reactive oxygen species. Redox Biol..

[14] Gutmann C., Siow R., Gwozdz A.M., Saha P., Smith A. (2020). Reactive Oxygen Species in Venous Thrombosis. Int. J. Mol. Sci..

[15] Mihara M., Uchiyama M. (1983). Effects of antioxidants on the TBA reaction of various rat liver homogenates. Biochem. Med..

[16] Yoshikawa T., Tanaka H., Kondo M. (1985). The increase of lipid peroxidation in rat adjuvant arthritis and its inhibition by superoxide dismutase. Biochem. Med..

[17] Jammes Y., Steinberg J.G., Mambrini O., Brégeon F., Delliaux S. (2005). Chronic fatigue syndrome: Assessment of increased oxidative stress and altered muscle excitability in response to incremental exercise. J. Intern. Med..

[18] Gris J.C., Quéré I., Sanmarco M., Boutiere B., Mercier E., Amiral J., Hubert A.M., Ripart-Neveu S., Hoffet M., Tailland M.L. (2000). Antiphospholipid and antiprotein syndromes in non-thrombotic, non-autoimmune women with unexplained recurrent primary early foetal loss. The Nîmes Obstetricians and Haematologists Study--NOHA. Thromb. Haemost..

[19] Sanmarco M., Bardin N., Camoin L., Beziane A., Gamerre M., Porcu G., Dignat-George F. (2007). Antigenic Profile, Prevalence, and Clinical Significance of Antiphospholipid Antibodies in Women Referred for in Vitro Fertilization. Ann. N. Y. Acad. Sci..

[20] Esmon N.L., Safa O., Smirnov M.D., Esmon C.T. (2000). Antiphospholipid Antibodies and the Protein C Pathway. J. Autoimmun..

[21] Smirnov M.D., Safa O., Regan L., Mather T., Stearns-Kurosawa D.J., Kurosawa S., Rezaie A.R., Esmon N.L., Esmon C.T. (1998). A Chimeric Protein C Containing the Prothrombin Gla Domain Exhibits Increased Anticoagulant Activity and Altered Phospholipid Specificity. J. Biol. Chem..

[22] Smirnov M.D., Esmon C.T. (1994). Phosphatidylethanolamine incorporation into vesicles selectively enhances factor Va inactivation by activated protein C. J. Biol. Chem..

